# Peptide Nucleic Acids: Applications in Biomedical Sciences

**DOI:** 10.3390/molecules25153317

**Published:** 2020-07-22

**Authors:** Eylon Yavin

**Affiliations:** The Institute for Drug Research, School of Pharmacy, Faculty of Medicine, Hebrew University of Jerusalem, Hadassah Ein Kerem, Jerusalem 9112102, Israel; eylony@ekmd.huji.ac.il; Tel.: +972-2-6758692

The DNA mimic, PNA (peptide nucleic acid), has been with us now for almost 3 decades. In the early 1990s, scientists from Denmark, led by Prof. Peter Nielsen [1,2], invented a very clever DNA analog that replaced the entire sugar–phosphate backbone in DNA with a neutral backbone that consisted of glycine–ethylenediamine (aeg = N-(2-aminoethyl) glycine). This analog was found to have much higher binding affinity to complementary DNA and RNA than natural DNA [2]. In addition, PNA was found to be highly stable in biological fluids [3].

Another critical issue that has translated PNA molecules into the biomedical field relates to the chemistry used to synthesize PNA oligomers; namely, solid phase peptide chemistry. This mode of synthesis led to a simple method to install cell permeation to PNA by attaching cell-penetrating peptides (CPPs) to either C or N termini of the PNA oligomer [4,5,6,7,8]. In addition, since aegPNAs have been invented, many chemical modifications to the basic aegPNA structure have been introduced. For example, fluorine-modified [9], cyclopentyl-modified [10], mini-peg-modified [11], guanidinium-modified [12], pyrrolidinyl-modified (acpc) [13] and 2-aminopyridine-modified [14] PNAs are just a few examples for chemical modifications that have led to a variety of improvements such as cell permeability, higher DNA or RNA binding affinity, and DNA duplex strand invasion (Scheme 1). Some of these modifications are highlighted in this current Special Issue.

Indeed, the introduction of a mini-peg at the gamma position of the PNA backbone has been shown to generate PNAs as powerful triplex-forming oligonucleotides (TFOs). This property has been used for gene editing in-vitro and in-vivo [15]. In this Special Issue, Economos et al. [16] review gene editing using a variety of chemically modified PNA (e.g., bisPNA, tail-clamp(tc)PNA, and gamma(γ)PNA) highlighting the clear potential of using this technology to treat monogenic disorders such as β-thalassemia.

The detection of minute amounts of mutated DNA (SNP-single nucleotide polymorphism) in a background of abundant wild type DNA is a formidable task. Such SNPs as those found in the KRAS and EGFR genes are associated with a variety of cancers and their detection may lead to early diagnosis of cancer with improved chances of recovery and overall survival. Fouz and Appella [17] review this area in relation to using PNA molecules as clamps that provide an approach to amplify mutated DNA by PCR in a highly specific manner. Here too, chemical modifications may be introduced to the PNA clamp (e.g., l or d Glu at the γ position) in order to increase or decrease binding affinity to the DNA strands [18].

Chemical modifications to PNA may be also introduced at either the C or N termini. The Kaihatsu lab [19] report in this Special Issue a panel of Tolane-modified PNAs (introduced at the N-terminus) that present good mismatch discrimination between single point mutated vs. wild type DNA or RNA. One such analog (a naphtyl derivative) was shown as a practical PNA probe for SNP detection of the influenza A virus neuraminidase gene that is associated with drug resistance.

PNA probes may be also used for the detection of specific RNA sequences. These RNAs may be in the form of mRNAs [20], lncRNAs [21], miRNAs, and others. An excellent review in this Special Issue, reported by Cadoni et al. [22], describes the various methodologies used in conjunction with PNA to detect miRNAs. Changes in miRNA expression are associated with a variety of diseases and therefore these miRNAs are considered as ideal disease biomarkers. However, the levels of miRNA in cells and especially in serum are extremely low. In this aspect, the authors highlight a variety of approaches that allow the detection of miRNAs initiated by PNA hybridization that is coupled to signal amplification.

The high affinity of PNA to complementary DNA and the achiral nature of aegPNA is also exploited for other diagnostic purposes. In this Special Issue, the Sczepanski group [23] report an l-DNA amplifier circuit capable of detecting native d-oligonucleotides. An important feature in this Catalytic Hairpin Assembly (HCA) circuit is that it is stable in serum.

The basic idea of using DNA and its analogs as antisense-based drugs was first reported in the late 70’s [24]. As of today, there are several approved drugs in the market that are based on antisense therapy [25]. PNA molecules are limited as classical antisense molecules due to the fact that they do not recruit RNAse H when bound to complementary RNA. Thus, PNAs are typically used as RNA “blockers” and not as RNA “degraders”.

In this Special Issue, two research groups report studies that use this property of PNAs as steric blockers for treating genetic disorders. The Gang Chen group report the effect of PNA in promoting exon inclusion related to Tauopathies [26]. In cell culture, PNA–neamine conjugates restored exon 10 inclusion levels (in the *MAPT* gene) to around 50%.

Shaiq Sultan et al. [27] report on the therapeutic potential of PNAs to treat Cystic Fibrosis (CF). The authors present data where PNA masking of miR-145-5p binding sites (that are present within the 3′UTR of the CFTR (Cystic Fibrosis Transmembrane Conductance Regulator) mRNA) are able to increase the expression of the miR-145-5p regulated CFTR that is repressed in this disease.

PNAs as antisense molecules have been shown to downregulate genes not only in mammalian cells, but also as antiviral, antibacterial, and antimalarial agents. In the final contribution to this Special Issue, Monika Wojciechowska et al. provide a comprehensive review on the various approaches used to develop PNA molecules as antibacterial agents [28].

The authors provide a detailed description on the various chemical modifications installed into the PNA as well as a variety of peptides used as shuttles for bacterial uptake of these PNA antibacterial agents. Given the growth in antibiotic resistance, there is indeed much room for developing such PNA antisense molecules that target critical genes in bacteria as a novel approach to provide antibacterial activity with minimal bacterial drug resistance.

In summary, this Special Issue manifests the variety of biomedical fields where PNA plays a critical role in diagnostics and therapeutics. The morpholino oligomer (phosphorodiamidate morpholino oligomer (PMO)), which is also a DNA mimic with a neutral backbone, has been approved by the FDA for treating Duchenne Muscular Dystrophy (DMD) by promoting exon skipping in the Dystrophin gene [25]. Given these developments and the advances in PNA chemistry, it still remains to be seen whether PNA will turn one day into an approved drug.

## Figures and Tables

**Scheme 1:**
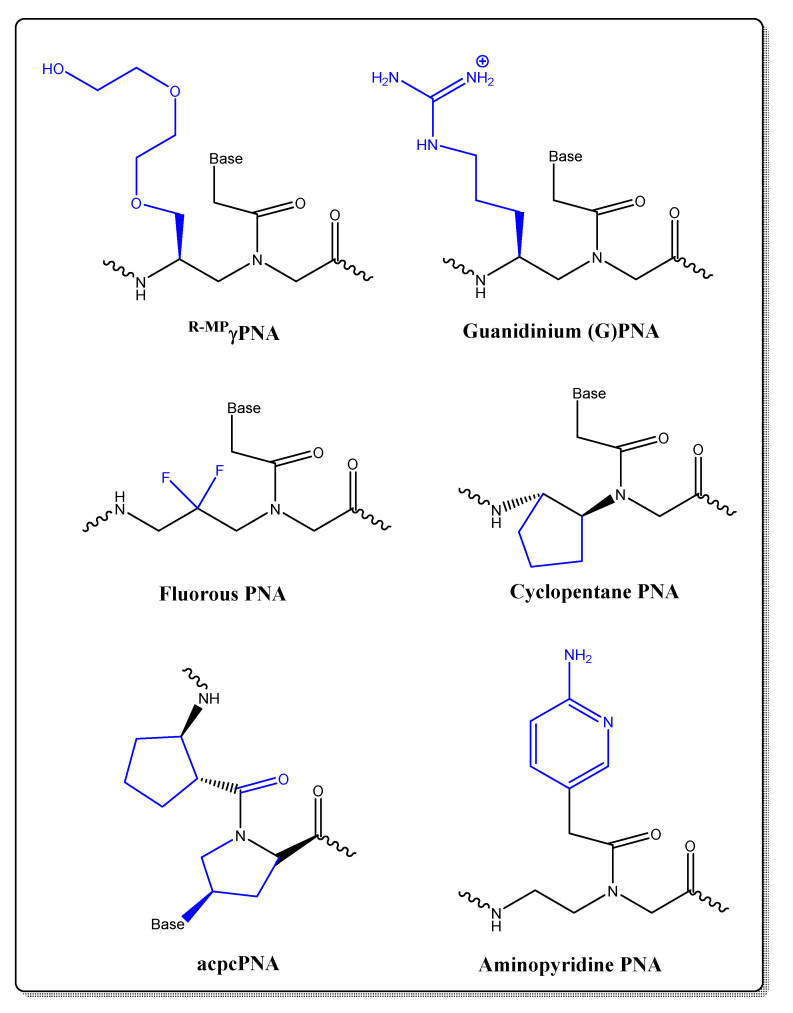
Chemical modifications on aegPNA.
